# Suitable methods of measuring acceleration time in the diagnosis of internal carotid artery stenosis

**DOI:** 10.1007/s10396-019-01000-x

**Published:** 2020-01-07

**Authors:** Kentaro Iizuka, Hidehiro Takekawa, Akio Iwasaki, Haruki Igarashi, Keisuke Suzuki, Saro Kobayashi, Daisuke Tsukui, Koichi Hirata

**Affiliations:** 1 Department of Neurology, Dokkyo Medical University, Tochigi, Japan; 2 Stroke Center, Dokkyo Medical University, 880 Kitakobayashi, Mibu, Tochigi 321-0293 Japan; 3 Center of Medical Ultrasonics, Dokkyo Medical University, Tochigi, Japan

**Keywords:** Acceleration time, Peak systolic velocity, Internal carotid artery stenosis, Measurement, Pulsed Doppler waveform

## Abstract

**Purpose:**

To enhance the utility of acceleration time (AcT) in the diagnosis of internal carotid artery (ICA) stenosis, we assessed the value of AcT measurements with different waveform patterns.

**Methods:**

Ninety-three patients with acute atherothrombotic cerebral infarction were enrolled, and they underwent both carotid ultrasonography and digital subtraction angiography (DSA). AcT was determined by a conventional procedure (using the first peak point or the bending point) and the peak systolic velocity (PSV) procedure. The AcT ratio was calculated as (AcT of ICA)/(AcT of the ipsilateral common carotid artery). We evaluated the correlation of stenosis rate as assessed by the North American Symptomatic Carotid Endarterectomy Trial method using DSA (DSA-NASCET) with the AcT of ICA (ICA-AcT), the AcT ratio measured by the conventional procedure (conventional AcT ratio), and the AcT ratio measured by the PSV procedure (PSV AcT ratio). The area under receiver operating characteristic curves (AUC) for DSA-NASCET was calculated based on the ICA-AcT and AcT ratio.

**Results:**

Forty-five vessels had 50% or greater ICA stenosis. DSA-NASCET was positively correlated with the conventional AcT ratio (*r* = 0.723), conventional ICA-AcT (*r* = 0.638), and PSV AcT ratio (*r* = 0.245). The corresponding AUCs for ICA stenosis ≥ 50% were 0.971, 0.886, and 0.572, respectively.

**Conclusion:**

We demonstrated the usefulness of the conventional procedure for diagnosing stenosis of ICA origin using AcT and showed that the AcT ratio was a more beneficial parameter than AcT.

## Introduction

Carotid artery ultrasonography is capable of noninvasive evaluation of the internal carotid artery (ICA). The peak systolic velocity (PSV) [1, 2], as well as the ICA PSV/CCA PSV, which is the ratio of the ICA PSV and PSV of the common carotid artery (CCA) [3], are widely used in the diagnosis of ICA stenosis.

In addition, the acceleration time (AcT) of the ICA, which can be measured by pulsed-wave Doppler, has been reported useful in cases, where measurement of the PSV of stenosis is difficult [4–8]. The AcT is prolonged in the presence of aortic valve stenosis (AS) [9]. However, the AcT ratio, which is the ratio of the AcT of ICA (ICA-AcT) to the ipsilateral AcT of CCA (CCA-AcT), is not affected by AS [10]. Therefore, the AcT ratio is estimated to be of higher diagnostic value than the AcT.

The systolic pulsed Doppler waveform is different in each case. The commonly observed waveform has a monomodal or bimodal peak pattern, where the time from minimum flow velocity, namely initiation of upstroke, to the PSV (time-to-PSV) is consistent with the acceleration time, and this time-to-PSV has often been defined as AT [11]. However, there is also another pattern presenting with a bending point in the monomodal peak pattern.

There is a study comparing digital subtraction angiography (DSA) with the ICA-AcT, in which the time from initiation of the upstroke to the first peak of the bimodal peak pattern was considered to be the AcT, and the time from initiation of the upstroke to the bending point in the monomodal peak pattern was considered to be the ICA-AcT [8]. In the case of a monomodal peak pattern with a bending point, detailed observation of the pulsed Doppler waveform is required, because there are concerns that these will be observed as a monomodal peak pattern without a bending point and the time-to-PSV could be diagnosed as AcT.

This is why it was considered easier to define the time-to-PSV as the AcT. Furthermore, there have been few reports looking at the relationship between stenosis rate on DSA and AcT [7, 8].

In the present study, we evaluated the usefulness of the time-to-PSV, being defined as AcT, in the diagnosis of stenosis using DSA, and we compared it to previous methods.

## Methods

Our subjects were 93 consecutive patients (mean age: 70.8 years, SD: ± 8.31, 73 men) who were hospitalized in the Dokkyo Medical University Hospital for acute atherothrombotic cerebral infarction and evaluated by both carotid artery ultrasonography and DSA between April 1, 2015 and March 31, 2018.

In 68 vessels, pulsed Doppler waveform patterns of the CCA and ipsilateral ICA were identical, showing a bimodal peak pattern without a bending point in which the initial peak of the wave coincides with the PSV. Therefore, only time-to-PSV was calculated as AcT, and those vessels were excluded. In addition, 20 vessels were excluded, because the ICA was obstructed at its origin. Ultimately, 98 vessels were retrospectively analyzed.

### Carotid artery ultrasonography and evaluation of stenosis

KI, HT, AS, YT, HI, MO, and AI performed carotid artery ultrasonography using the SSA-770A (TOSHIBA, Japan), TUS-AI800, and TUS-A500 (CANON MEDICAL SYSTEMS Corporation, Tochigi, Japan).

Carotid artery ultrasonography was performed, while the subject was in the supine position, with the head turned away from the scanned side and the neck slightly extended.

The pulsed Doppler waveform of the CCA was measured approximately 2 cm from the carotid sinus using a linear-array probe (5–11 MHz, 4.0–11.0 MHz, and 3.5–11.5 MHz). The pulsed Doppler waveform of the ICA was measured approximately 3.5 cm (3.56 ± 1.00 cm) from the origin of the ICA using a convex-array probe (1.9–6.1 MHz, 1.5–6.0 MHz, and 1.8–6.2 MHz).

The sample volume of the pulsed-wave Doppler was set at 1/2 or 2/3 of the vessel diameter, and the Doppler insonation angle against the direction of blood flow was 60° or smaller. Sweep speed was fixed at 43.8 mm/sec (SSA-770A and TUS-A500) and 60.0 mm/sec (TUS-AI800).

The measurement was continued for five beats or more, until the same waveform was plotted by the pulsed-wave Doppler.

The stenosis ratio of the ICA origin diagnosed via DSA was evaluated by DT, SK, and TN using the North American Symptomatic Carotid Endarterectomy Trial method (DSA-NASCET) [12].

### Evaluation of acceleration time

**Fig. 1:**
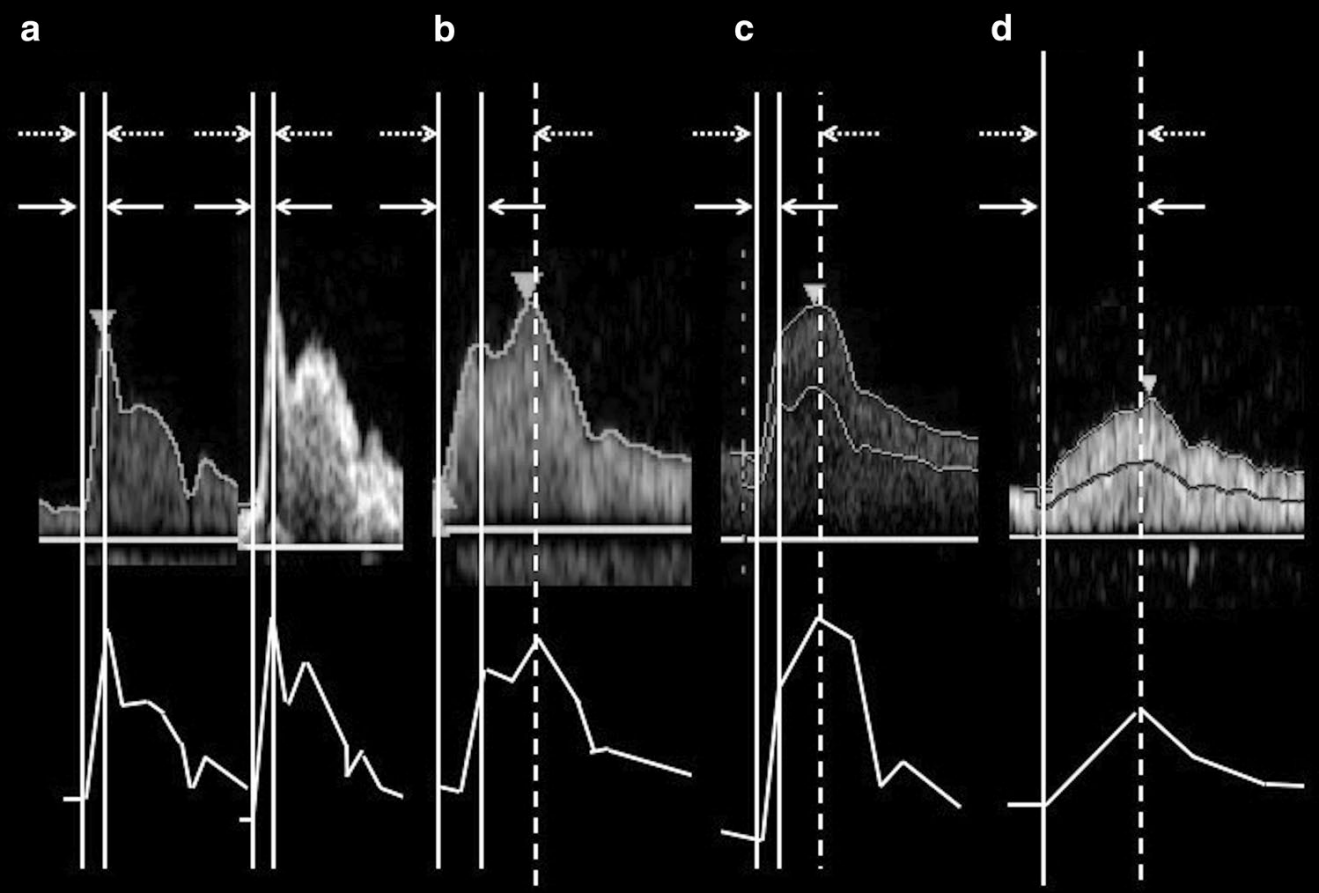
Measurements of conventional AcT and PSV AcT. “Conventional AcT” measurement (the time between the solid lines) and PSV AcT measurement (the time between the dotted lines). **a** Type A: This is a bimodal peak pattern; the initial peak of the waveform is consistent with PSV. **b** Type B: This is a bimodal peak pattern; the second peak of the waveform is consistent with PSV. **c** Type C: This is a monomodal peak pattern; the waveform has a clear bending point. **d** Type D: This is a monomodal peak pattern, where the bending point is unclear. *AcT* acceleration time, *PSV* peak systolic velocity

Pulsed Doppler waveforms were classified into the following four types (Fig. 1):

- Type A) A bimodal peak pattern, where the initial peak of the wave coincides with the PSV;
- Type B) A waveform, where the second peak coincides with the PSV of the bimodal peak pattern;
- Type C) A waveform of a monomodal peak pattern and a clear bending point; and
- Type D) A monomodal peak pattern without a clear bending point.

We measured “conventional AcT” of the CCA (“conventional CCA-AcT”) and ICA (“conventional ICA-AcT”) according to Takekawa’s method [5]. Briefly, for a bimodal pattern, the time from initiation of upstroke to the first maximum peak of the waveform was defined as “conventional AcT”, and for a monomodal peak pattern with a distinct bending point, the time to the bending point was defined as “conventional AcT” (Fig. 1, solid lines). We also defined the time-to-PSV as the “PSV AcT” and measured the CCA and ICA as “PSV CCA-AcT” and “PSV ICA-AcT”. Therefore, the “conventional AcT” and the “PSV AcT” coincided for the Type A and Type D patterns, and for the Type B and Type C patterns, the “PSV AcT” was more extended than the “conventional AcT”.

The AcT ratio, which is the ratio of the ICA-AcT to the CCA-AcT, was calculated.

In other words, the “conventional AcT ratio” was derived from the “conventional ICA-AcT”/“ipsilateral conventional CCA-AcT”. At the same time, the “PSV AcT ratio” was derived from the “PSV ICA-AcT”/ipsilateral “PSV CCA-AcT”.

The AcT used an average of five heartbeats, and the AcT and AcT ratio were measured by KI, HT, and AI, without taking into account the DSA-NASCET results.

### Statistical analysis

We evaluated the correlation of the DSA-NASCET with the “conventional ICA-AcT”, “PSV ICA-AcT”, “conventional AcT ratio”, and “PSV AcT ratio”, using the Pearson correlation coefficient. Then, we evaluated whether the DSA-NASCET was predictable for each item, using single regression analysis. We also calculated the area under the curve (AUC) based on the “conventional ICA-AcT”, “PSV ICA-AcT”, “conventional AcT ratio”, and “PSV AcT ratio”, using a receiver operating characteristic (ROC) curve.

We calculated the inter-rater reliability for the AcT and the DSA-NASCET of five vessels, using the intraclass correlation coefficients (ICC).

IBM SPSS (ver. 24.0, Tokyo, Japan) was used for statistical processing and plotting, and *p* < 0.05 was considered statistically significant.

HT, KS, and HK were involved in overseeing the entire study.

## Results

### All vessels

The ICCs for the “conventional ICA-AcT”, “PSV ICA-AcT”, and DSA-NASCET were 0.714, 0.994, and 0.998, respectively.

The CCA pulsed Doppler waveforms were Type A, 87 vessels; Type B, 8 vessels; Type C, 2 vessels; and Type D, one vessel. The ICA waveforms were Type B, 39 vessels; Type C, 41 vessels; and Type D, 18 vessels, and there was no Type A (Table 1).

**Table 1:** Types of pulsed Doppler waveform of the common carotid artery and internal carotid artery

Pulsed Doppler waveform type of CCA		Pulsed Doppler waveform type of ICA	
Type A (vessels)	87	Type A (vessels)	0
		Type B (vessels)	37
		Type C (vessels)	34
		Type D (vessels)	16
Type B (vessels)	8	Type A (vessels)	0
		Type B (vessels)	0
		Type C (vessels)	7
		Type D (vessels)	1
Type C (vessels)	2	Type A (vessels)	0
		Type B (vessels)	1
		Type C (vessels)	0
		Type D (vessels)	1
Type D (vessels)	1	Type A (vessels)	0
		Type B (vessels)	1
		Type C (vessels)	0
		Type D (vessels)	0

Type A was most commonly seen in CCA, and Type B was most commonly seen in ICA

*CCA* common carotid artery, *ICA* internal carotid artery

The median DSA-NASCET was 38.6% (range 0–85.2); stenosis ≥ 50% was identified in 45 subjects (45.9%), and of these, 29 subjects had stenosis of ≥ 70%.

DSA-NASCET showed significant correlation with the “conventional ICA-AcT” (*p* < 0.0001), “conventional AcT ratio” (*p* < 0.0001), and “PSV AcT ratio” (*p* = 0.0150), but no correlation was identified for “PSV ICA-AcT” (*p* = 0.629).

**Fig. 2:**
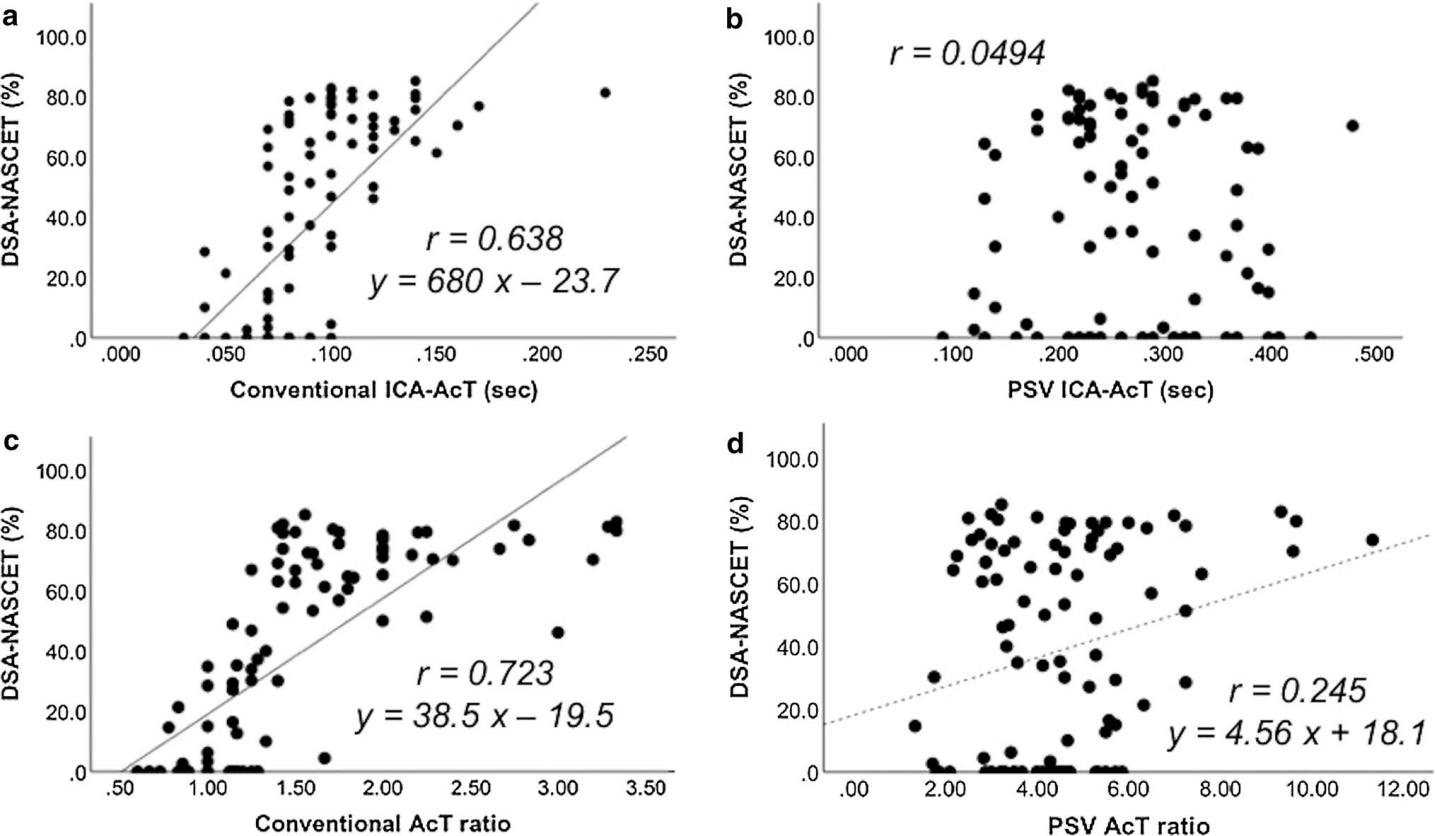
Correlations between AcT, AcT ratio, and DSA-NASCET. Although a significant positive correlation was found in single regression analysis with **a** “conventional ICA-AcT” (*r* = 0.638), **c** “conventional AcT ratio” (*r* = 0.723), and **d** “PSV AcT ratio” (*r* = 0.245), there was no correlation for **b** “PSV ICA-AcT”. *AcT* acceleration time, *PSV* peak systolic velocity, *ICA* internal carotid artery

Figure 2 shows the results of a single regression analysis. The correlation coefficient of the “conventional ICA-AcT” was 0.638, and the “conventional AcT ratio” was 0.723, so the latter exhibited a greater correlation. The correlation coefficient of the “PSV AcT ratio” exhibited a low value of 0.245.

**Fig. 3:**
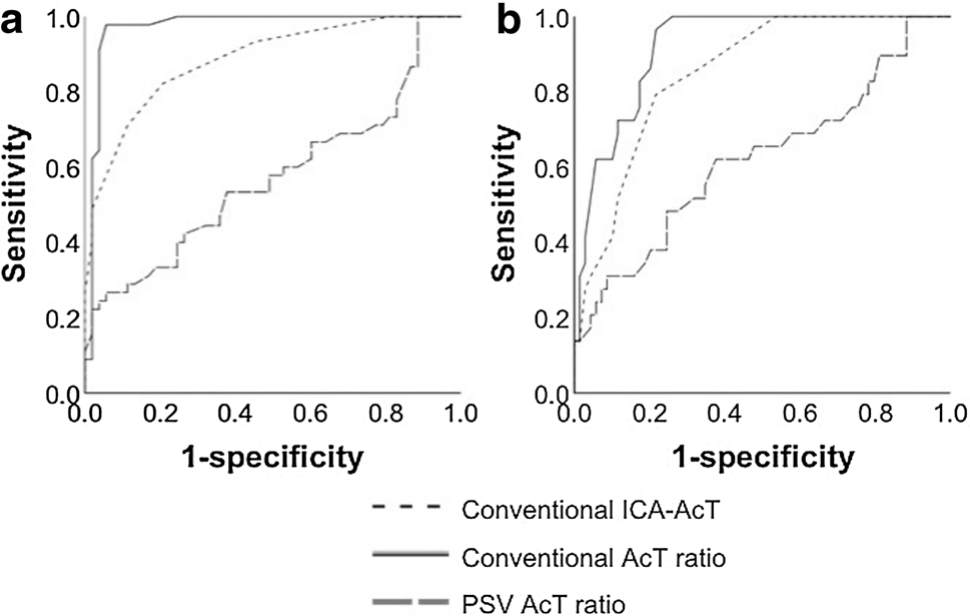
ROC curve for diagnosis of DSA-NASCET. For predicting DSA-NASCET ≥ 50% on the ROC curve, the “conventional AcT ratio” was 0.971, the “conventional ICA-AcT” was 0.886, and the “PSV AcT ratio” was 0.572 (**a**). For predicting DSA-NASCET ≥ 70% on the ROC curve, the “conventional AcT ratio” was 0.920, the “conventional ICA-AcT” was 0.852, and the “PSV AcT ratio” was 0.621 (**b**). *ROC* receiver operating characteristic, *DSA* digital subtraction angiography, *NASCET* north american symptomatic carotid endarterectomy trial method, *AcT* acceleration time, *PSV* peak systolic velocity, *ICA* internal carotid artery, *AUC* area under the curve

The AUC of the ROC curve at DSA-NASCET ≥ 50% had a “conventional AcT ratio” of 0.971, and a “conventional ICA-AcT” of 0.886, thus exhibiting a high degree of utility in the diagnosis of stenosis. However, the AUC of the “PSV AcT ratio” was low at 0.572 (Fig. 3a). The results of the AUC of DSA-NASCET ≥ 70% presented a “conventional AcT ratio” of 0.920, “conventional ICA-AcT” of 0.852, and “PSV AcT ratio” of 0.621, thus being similar to the DSA-NASCET ≥ 50% stenosis results (Fig. 3b).

### 11 blood vessels, where the CCA pulsed Doppler waveform types were B, C, or D

The pulsed Doppler waveform types for the ICA were: Type B, 2 vessels; Type C, 7 vessels; and Type D, 2 vessels.

The DSA-NASCET median was 50.0% (range 0–66.7), and six vessels (54.5%) were ≥ 50%.

In relation to DSA-NASCET, although a significant correlation was seen with the “conventional AcT ratio” (*p* = 0.00344), there was no correlation with the “conventional ICA-AcT” (*p* = 0.0722), “PSV ICA-AcT” (*p* = 0.101), and “PSV AcT ratio” (*p* = 0.903), whereas the correlation coefficient with the “conventional AcT ratio” was 0.795 and the AUC was 1.00.

## Discussion

The results of investigation of the AcT measurement method, used in diagnosis of the ICA origin stenosis, indicated that the “conventional AcT” method, where the time from initiation of upstroke to the first peak or the bending point of the systolic velocity was treated as the AcT, was more useful than the “PSV AcT”, where the time-to-PSV was treated as the AcT. In addition, the “conventional AcT ratio” exhibited a more useful effect compared to the “conventional ICA-AcT”.

There are few reports on the diagnosis of ICA origin stenosis, where the AcT or the AcT ratio was used [4–8]. Takekawa et al. [4] reported on 127 blood vessels, where the PSV and the ICA-AcT exhibited a significant positive correlation. Tamura et al. [6] evaluated 266 blood vessels using carotid artery ultrasonography. Having used the same stenosis rate measurement method as the DSA-NASCET, the ICA-AcT was useful in the diagnosis of the stenosis rate. In addition, the AcT ratio was also useful for ICA origin stenosis. Takekawa et al. [5] evaluated the “conventional AcT ratio”. They studied its relationship with the diameter stenosis rate evaluated by carotid artery ultrasonography, and reported that there was a significant positive correlation between both; this suggested that the diameter stenosis rate is ≥ 50% when the conventional AcT ratio exceeds 1.5.

On the other hand, Kamiya et al. [7] and Nishihira et al. [8] studied the relationship between DSA-NASCET and AcT. Kamiya et al. [7] reported that the ICA-AcT and the AcT ratio were useful in the diagnosis of restenosis, using DSA or 3D CT angiography on patients who had undergone carotid artery stenting. Nishihira et al. [8] measured the DSA-NASCET, “conventional AcT”, and AcT ratio of 177 blood vessels and studied their relationship. Their results showed that the DSA-NASCET could be ≥ 50% if the “conventional AcT ratio” exceeded 1.3.

It is worth mentioning that though an echocardiogram was obtained for each of the 47 cases in this study, severe AS was diagnosed in only one case. The subjects in this group were free of any valvular heart disease, or if present, it was trivial or mild, and there were no other cardiac diseases present. This is the reason why studies on cardiac diseases, including valvular heart disease, are difficult to perform. However, the subject with severe AS exhibited left ICA occlusion and only 6.2% stenosis of the right ICA at DSA-NASCET. The “conventional ICA-AcT” was 0.7 s in this case, the “PSV ICA-AcT” was extended at 0.24 s, and though the “PSV AcT ratio” exhibited a high value of 3.43, the “conventional AcT ratio” was 1.00. Thus, AS causes AcT elongation [9], and it is possible that the AcT ratio can exclude the effect of AS. Actually, Okamura et al. [10] studied the effects of AS, aortic regurgitation (AR), and ejection fraction (EF) on the AcT ratio on 94 blood vessels, and reported that the AcT ratio was not affected by age and sex, and neither were AS, AR, and EF.

Although these past reports suggested that AcT and the AcT ratio were useful in the diagnosis of stenosis of ICA origin, there was still a variance with the ICA-AcT cutoff values of 85–150 ms [4, 6, 8], and AcT ratios of 1.31–1.75 [4, 5, 8], even in stenosis rates considered to be DSA-NASCET ≥ 50%.

One of the possible reasons for the difference in the cutoff values across the reports may have been the existence of multiple types of pulsed Doppler waveforms, and this fact influenced the AcT measurements, resulting in different reported values.

Sung et al. [13] studied factors affecting the pulsed Doppler waveform using a closed-circuit in vitro blood flow model. They reported that in cases, where the vascular compliance at the distal and the proximal locations of pulsed Doppler waveform measurement was low, it rapidly reached a peak in the initial systolic period, and also presented a mild bimodal peak pattern. In addition, various patterns appeared by changing the vascular compliance. Suzuki et al. [14] studied the pulsed Doppler waveforms of 32 blood vessels, and their results showed that about 25% did not present as a type A waveform. They reported that the AR, the resistance index, the time-averaged maximum flow velocity, and the end diastolic velocity exerted an effect on the pulsed Doppler waveform pattern. In addition, Fujishiro and Yoshimura [15] studied the relationship of age with the CCA pulsed Doppler waveform in 20 healthy subjects, using an ultrasonic quantitative flow measurement system. Although vessel diameters grew thicker with age, deviations of vessel diameter, mean blood flow velocity, and mean blood flow volume became smaller. Although the first peak of the systole wave was sharp and high in young people, this tendency became less obvious with age.

The anacrotic notch [16] (AN) presents the maximal point of the arterial blood flow velocity, and the dicrotic notch (DN) reflects the opening and closing of the aortic valve [17]. In peripheral arteries, the AN is affected by hardening of the vascular walls [15] and by a high systolic pulmonary arterial pressure value [18]. The DN, in addition to its correlation with central aortic blood pressure [19], is affected by the EF [20], peripheral artery resistance [21], and aortic valve disease [17]. In another study, Okabe et al. [22] evaluated 37 cases and reported that the DN became unclear with advanced age, a high plaque score, and low PSV.

“PSV ICA-AcT” and “PSV AcT ratio” may have low utility as the pulsed Doppler waveform changes under the influence of various factors, such as age, heart disease, and arteriosclerosis. The results of our study point to the possibility that the “conventional AcT”, especially “conventional AcT ratio”, is a useful indicator for diagnosing stenosis of ICA origin in patients in whom measurements of the PSV of the maximum stenosis point and ICA PSV/CCA PSV are difficult.

Our study had several limitations. First, we excluded 68 vessels in which pulsed Doppler waveforms showed Type A patterns in both the CCA and ipsilateral ICA. Therefore, the usefulness of AcT in vessels that had identical patterns of “conventional AcT” and “PSV ICA-AcT” could not be evaluated. In addition, because only 11 vessels showed patterns of Type B, C, or D in the CCA, further evaluations for those patterns other than Type A are needed. Finally, pulsed Doppler waveforms were measured at a constant sweep speed, but a change in the sweep speed could result in different patterns in pulsed Doppler waveforms. Determining the optimal sweep speed for AcT measurements requires further studies.

## Conclusion

We studied the value of AcT measurement in stenosis of ICA origin. Our study demonstrated that the proper measurement method for AcT was the “conventional AcT” method, measuring the time from initiation of upstroke to the initial peak or to the bending point. According to our inferences, the “conventional AcT ratio” has a still greater utility compared with the “conventional ICA-AcT”. There are few reports, where DSA-NASCET and AcT are compared. Multicenter studies should be conducted in the future, where “conventional AcT” or the “conventional AcT ratio” and DSA-NASCET are studied. Guidelines for the diagnosis of stenosis of ICA origin need to be created based on the knowledge of its relationship to AcT or the AcT ratio.
